# PD194 The Scenario Of Hospital-Based Health Technology Assessment In Brazil, Italy, And Poland

**DOI:** 10.1017/S0266462324004161

**Published:** 2025-01-07

**Authors:** Rossella Di Bidino, Iga Lipska, Monika Kukla, Marilia Mastrocolla de Almeida Cardoso

## Abstract

**Introduction:**

Hospital-based health technology assessment (HB-HTA) is evolving constantly. In 2023, a survey was conducted by the HB-HTA Interest Group of Health Technology Assessment International with the aim of collecting data on HB-HTA activities, including perceptions of the role of, potential for, and barriers to HB-HTA. Overall, 87 responses were collected: 41 from hospitals performing health technology assessment (HTA), 18 from hospitals not conducting HTA, and 28 from policymakers.

**Methods:**

The survey collected data from 28 countries. Italy, Poland, Brazil provided the highest number of responses. We conducted a descriptive comparative analysis focusing on these three countries to investigate whether and how the policies for HB-HTA, the activities of HB-HTA, and the perceptions of HB-HTA differed among policymakers and hospitals not performing HTA.

**Results:**

Overall, 19 responses were collected from Italy and 10 each were collected from Brazil and Poland. While Italy (n=10) and Brazil (n=8) had a high number of responses from hospitals performing HB-HTA, most of the responses from Poland (n=9) were from hospitals not performing HTA. There was a lack of policies for HB-HTA in all three countries. HTA was performed in big hospitals (≥500 beds) in Italy and Brazil. HB-HTA covered all kinds of technologies, including digital health. In Poland, hospitals not conducting HTA recognized the ability of HB-HTA to promote cost containment. Policymakers were open to including HB-HTA in their activities.

**Conclusions:**

Interpreting the diffusion of HB-HTA, its integration with other HTA processes, and the perceptions of its pros and cons should be both country specific and be conducted from an international perspective. The analysis demonstrated the need for specific policies and for better dissemination and promotion of HB-HTA activities and collaborations with HTA agencies.

